# Prognostic usefulness of a modified risk model for solitary fibrous tumor that includes the Ki-67 labeling index

**DOI:** 10.1186/s12957-022-02497-2

**Published:** 2022-02-01

**Authors:** Shintaro Sugita, Keiko Segawa, Noriaki Kikuchi, Tomoko Takenami, Tomomi Kido, Makoto Emori, Yukinori Akiyama, Kohichi Takada, Shiro Hinotsu, Tadashi Hasegawa

**Affiliations:** 1 Department of Surgical Pathology, Sapporo Medical University, School of Medicine, Sapporo, Hokkaido 060-8543 Japan; 2 Department of Surgical Pathology, Sunagawa City Medical Center, Sunagawa, Hokkaido 073-0196 Japan; 3 Department of Orthopedic Surgery, Sapporo Medical University, School of Medicine, Sapporo, Hokkaido 060-8543 Japan; 4 Department of Neurosurgery, Sapporo Medical University, School of Medicine, Sapporo, Hokkaido 060-8543 Japan; 5 Department of Medical Oncology, Sapporo Medical University, School of Medicine, Sapporo, Hokkaido 060-8543 Japan; 6 Department of Biostatistics and Data Management, Sapporo Medical University, School of Medicine, Sapporo, Hokkaido 060-8543 Japan

**Keywords:** Solitary fibrous tumor, Risk model, STAT6, Ki-67 labeling index

## Abstract

**Background:**

Predicting the prognosis of patients with solitary fibrous tumor (SFT) is often difficult. The prognostic risk models developed by Demicco et al. are now the standard for evaluating the risk of SFT metastasis in the current World Health Organization classification of soft tissue and bone tumors.

**Methods:**

In this study, we examined the prognostic usefulness of a modified version of the Demicco risk models that replaces the mitotic count with the Ki-67 labeling index. We compared the three-variable and four-variable Demicco risk models with our modified risk models using Kaplan–Meier curves based on data for 43 patients with SFT.

**Results:**

We found a significant difference in metastasis-free survival when patients were classified into low-risk and intermediate/high-risk groups using the three-variable (*P* = 0.022) and four-variable (*P* = 0.046) Demicco models. There was also a significant difference in metastasis-free survival between the low-risk and intermediate/high-risk groups when the modified three-variable (*P* = 0.006) and four-variable (*P* = 0.022) models were used.

**Conclusion:**

Modified risk models that include the Ki-67 labeling index are effective for prediction of the prognosis in patients with SFT.

## Introduction

The current World Health Organization (WHO) classification of soft tissue and bone tumors defines solitary fibrous tumor (SFT) as a rarely metastasizing intermediate malignant tumor showing fibroblastic/myofibroblastic differentiation [1]. SFT usually affects adults, with a peak incidence between 40 and 70 years and no sex predilection. SFT can occur at any anatomical site, including the intrathoracic cavity (pleura and lung), intra-abdominal cavity (retroperitoneum and pelvis), central nervous system (meninges), extremities, head and neck, and trunk. SFT arises more frequently in deep soft tissue than in superficial soft tissue. Histologically, SFT is characterized by a patternless proliferation of bland spindle cells accompanied by collagenous stroma and hemangiopericytoma-like vessels. On immunohistochemistry (IHC), SFT is usually positive for CD34 and STAT6 [2]. Genetically, SFT has a specific *NAB2-STAT6* fusion gene [3, 4].

Predicting the prognosis of SFT is often difficult. The majority of SFTs have morphologically bland features and a benign clinical course. However, some SFTs with morphologically benign features have a fatal outcome. Therefore, it can be difficult to predict the prognosis of SFT based on histological parameters alone. Several risk models have been proposed for predicting the prognosis in patients with SFT and the system developed by Demicco et al. is becoming established as a standard for evaluation of metastatic risk [5, 6]. Their system has been accepted in the current World Health Organization (WHO) classification of soft tissue and bone tumors and contains three variables (patient age, tumor size, mitotic count) or four variables (addition of necrosis) for evaluation of metastatic risk in patients with SFT [1, 5, 6].

Sarcoma grading systems often contain mitotic count as one of the histological parameters. Evaluation of mitotic count is included in the classical Federation Nationale des Centres de Lutte le Cancer (FNCLCC) grading system, which is the most widely used grading method in the world [7]. Although evaluation of mitotic figures tends to differ among observers [8], we have developed a grading system that uses the Ki-67 labeling index (LI) instead of the mitotic count in order to provide a more universal grading system for predicting the prognosis of soft tissue sarcoma [9–12]. In this study, we clarified the prognostic usefulness of the modified risk models for SFT proposed by Demicco et al. [1, 5, 6] when the mitotic count is replaced by the Ki-67 LI.

## Materials and methods

### Sample selection

We identified 43 cases of SFT in the archives of the Department of Surgical Pathology, Sapporo Medical University Hospital (Hokkaido, Japan), Kushiro City General Hospital (Hokkaido, Japan), and Sunagawa City Medical Center (Hokkaido, Japan). We performed hematoxylin-eosin staining using 3-μm-thick sections from formalin-fixed and paraffin-embedded tumor tissues. We reviewed all hematoxylin-eosin slides for the individual cases and morphologically confirmed the diagnosis of SFT.

### Evaluation of clinicopathological parameters for risk models

We evaluated the risk factors in our cases according to the three-variable and four-variable risk models developed by Demicco et al. [5, 6]. These are the best known models for predicting the risk of metastasis of SFTs. These tumors are categorized as low risk, intermediate risk, and high risk by summing the scores for individual clinicopathological parameters. We assessed the maximum diameter of each tumor based on the macroscopic, histological, or radiological findings in the electronic medical records. Tumor necrosis was evaluated based on macroscopic findings and histological confirmation on the available slides. Degree of tumor necrosis was estimated according to whether the necrotic area occupied less than 10% of the tumor or more. Histologically, we recognized mitotic figures only by the number of tumor cells with divided nuclear chromatin indicating mitosis. We estimated the frequency of mitosis by counting the number of tumor cells per 1 mm^2^ field. The clinicopathological parameters in the three-variable Demicco model include patient age (score 0, < 55 years; score 1, ≥ 55 years), tumor size (score 0, 0–4.9 cm; score 1, 5–9.9 cm; score 2, 10–14.9 cm; score 3, ≥ 15 cm), and mitotic frequency of tumor cells (score 0, 0/mm^2^; score 1, 0.5–1.5/mm^2^; score 2, ≥ 2/mm^2^) with a total score of 0–2 points indicating low risk, 3–4 points indicating intermediate risk, and 5–6 points indicating high risk [5]. In the four-variable Demicco model, tumor necrosis (score 0, < 10%; score 1, ≥ 10%) is added to patient age, tumor size, and mitotic frequency of tumor cells (low risk, 0–3 points; intermediate risk, 4–5 points; and high risk, 6–7 points) [6].

### Immunohistochemistry

We reviewed all previously stained IHC slides and confirmed that the findings were consistent with a diagnosis of SFT. Next, we performed IHC for STAT6 and Ki-67 using representative sections from formalin-fixed and paraffin-embedded tissues in all cases. These tissues were sliced into 3-μm-thick sections and examined with an automated IHC system at Sapporo Medical University Hospital. All slides were loaded into a PT Link module (Agilent Technologies, Santa Clara, CA) and subjected to a heat-induced antigen-retrieval protocol with EnVision FLEX Target Retrieval Solution (Agilent) before being transferred to the Autostainer Link 48 instrument (Agilent). We used antibodies against STAT6 (rabbit polyclonal, dilution 1:1000; Santa Cruz Biotechnology, Dallas, TX) and Ki-67 (clone MIB-1, ready-to-use, FLEX; Dako, Carpinteria, CA). We estimated only the nuclear staining of these markers.

### Evaluation of Ki-67 LI by digital image analysis and definition of the modified Demicco risk model using the Ki-67 LI score

We first selected one area corresponding to a “hot spot” on a Ki-67-stained slide for analysis by optical microscopy. This “hot spot” consisted almost entirely of SFT cells without contamination from non-tumor cells, such as inflammatory infiltrates. We used a manually captured image (MCI) method that involved selection of a “hot spot” Ki-67 image with a microscope digital camera in real time at × 200 magnification and quantification of the MCI by image analysis software (Patholoscope; Mitani Corp., Tokyo, Japan), with the parameters set in advance for Ki-67 staining at our institution [13]. The count images for each case were reviewed to verify the accuracy of quantitation in the image analysis. In most cases, the expected results were obtained in one analytic step. We categorized the Ki-67 LI into three scores (0, < 1%; 1, 1–10%; 2, ≥ 10%). Moreover, we modified the three-variable and four-variable Demicco risk models using the Ki-67 LI score instead of the mitotic frequency score for the three-variable model, which included patient age (score 0, < 55 years; score 1, ≥ 55 years), tumor size (score 0, 0–4.9 cm; score 1, 5–9.9 cm; score 2, 10–14.9 cm; score 3, ≥ 15 cm), and Ki-67 LI score (0, < 1%; 1, 1–10%; 2, ≥ 10%) for low risk (0–2 points), intermediate risk (3–4 points), and high risk (5–6 points). The modified four-variable model included tumor necrosis (score 0, <10%; score 1, ≥ 10%) in addition to patient age, tumor size, and the Ki-67 LI score for low risk (0–3 points), intermediate risk (4–5 points) and high risk (6–7 points).

### Statistical analysis

The total duration of follow-up and time until distant metastasis were calculated from the date of surgical resection or biopsy. MFS was estimated by Kaplan–Meier curve analysis, and the log-rank test was used to assess differences between groups. All statistical analyses were performed using JMP Pro 15 (SAS Institute Inc., Cary, NC). For all analysis, differences at *P* < 0.05 were considered statistically significant.

## Results

### Clinical findings

The demographic and clinical characteristics of the 43 patients with SFT included in this study are summarized in Table 1. The patients comprised 21 men and 22 women of mean age 54.6 years (median 56, range 19–82). The anatomical locations were as follows: intrathoracic (lung, *n* = 7; pleura, *n* = 3), intra-abdominal (retroperitoneum, *n* = 4; urinary bladder, *n* = 3; pelvic cavity, *n* = 2; prostate, *n* = 1; pancreas, *n* = 1; peritoneum, *n* = 1), central nervous system (meninges, *n* = 5; spinal cord, *n* = 1), and other (extremity, *n* = 7; head and neck, *n* = 5; trunk, *n* = 3). The mean maximum tumor diameter was 6.4 cm (median 4.5, range 1.0–16.0). The mean follow-up duration was 63 months (median 43, range 1–250), at the end of which 32 patients were alive without local recurrence or metastasis. Five patients were alive with disease and six had died as a result of local recurrence and/or distant metastasis.

**Table 1:** Clinicopathological parameters included in risk models for solitary fibrous tumor

Variable	Patients, *n* (%)	5-year MFS (%) (95% CI)	Log-rank *p* value
Age (years)			
< 55 (score 0)	19 (44.2)	100 (^a^)	0.218
≥ 55 (score 1)	24 (55.8)	87.2 (60.2–96.8)	
Sex			
Male	21 (48.8)	85.6 (56.1–96.5)	0.121
Female	22 (51.2)	100 (^a^)	
Location			
IT	10 (23.3)	100 (^a^)	0.229
IA	12 (27.9)	87.5 (46.3–98.3)	
CNS	6 (14.0)	100 (^a^)	
Other	15 (34.9)	88.9 (50.0–98.5)	
Tumor size (cm)			
0–4.9 (score 0)	24 (55.8)	100 (^a^)	0.095
5–9.9 (score 1)	10 (23.3)	100 (^a^)	
10–14.9 (score 2)	3 (7.0)	100 (^a^)	
≥ 15 (score 3)	6 (14.9)	60.0 (20.0–90.0)	
Mitoses/mm^2^			
0 (score 0)	34 (79.1)	94.7 (70.6–99.3)	0.982
0.5–1.5 (score 1)	4 (9.3)	100 (^a^)	
≥ 2 (score 2)	5 (11.6)	66.7 (15.4–95.7)	
Ki-67 LI (%)			
< 1 (score 0)	15 (34.9)	90.0 (53.3–98.6)	0.018
1–10 (score 1)	24 (55.8)	100 (^a^)	
≥ 10 (score 2)	4 (9.3)	50.0 (5.8–94.1)	
Tumor necrosis (%)			
< 10 (score 0)	40 (93.0)	92.3 (73.5–98.1)	0.978
≥ 10 (score 1)	3 (7.0)	100 (^a^)	
Dedifferentiation			
Present	2 (4.7)	0	0.000
Absent	41 (95.3)	95.7 (74.8–99.4)	
Three-variable risk model (age, size, mitoses)			
Low (0–2 points)	33 (76.7)	100 (^a^)	0.049
Intermediate (3–4 points)	7 (16.3)	50.0 (5.9–94.1)	
High (5–6 points)	3 (7.0)	66.7 (15.4–95.7)	
Four-variable risk model (age, size, mitoses, necrosis)			
Low (0–3 points)	37 (86.0)	100 (^a^)	0.060
Intermediate (4–5 points)	4 (9.3)	50.0 (5.9–94.1)	
High (6–7 points)	2 (4.7)	50.0 (5.9–94.1)	
Modified three-variable model (age, size, Ki-67 LI)			
Low risk (0–2 points)	29 (67.4)	100 (^a^)	0.006
Intermediate/high risk (3–6 points)	14 (32.6)	74.1 (35.6–93.7)	
Modified four-variable model (age, size, Ki-67 LI, necrosis)			
Low risk (0–3 points)	35 (81.4)	100 (^a^)	0.022
Intermediate/high risk (4–7 points)	8 (18.6)	62.5 (21.9–90.9)	

*CI* confidence interval, *CNS* central nervous system, *IA* intra-abdominal, *IT* intrathoracic, *LI* labeling index, *MFS* metastasis-free survival

^a^95% CI was not calculated because of no events until 5 years

### Histological findings for solitary fibrous tumor

**Fig. 1:**
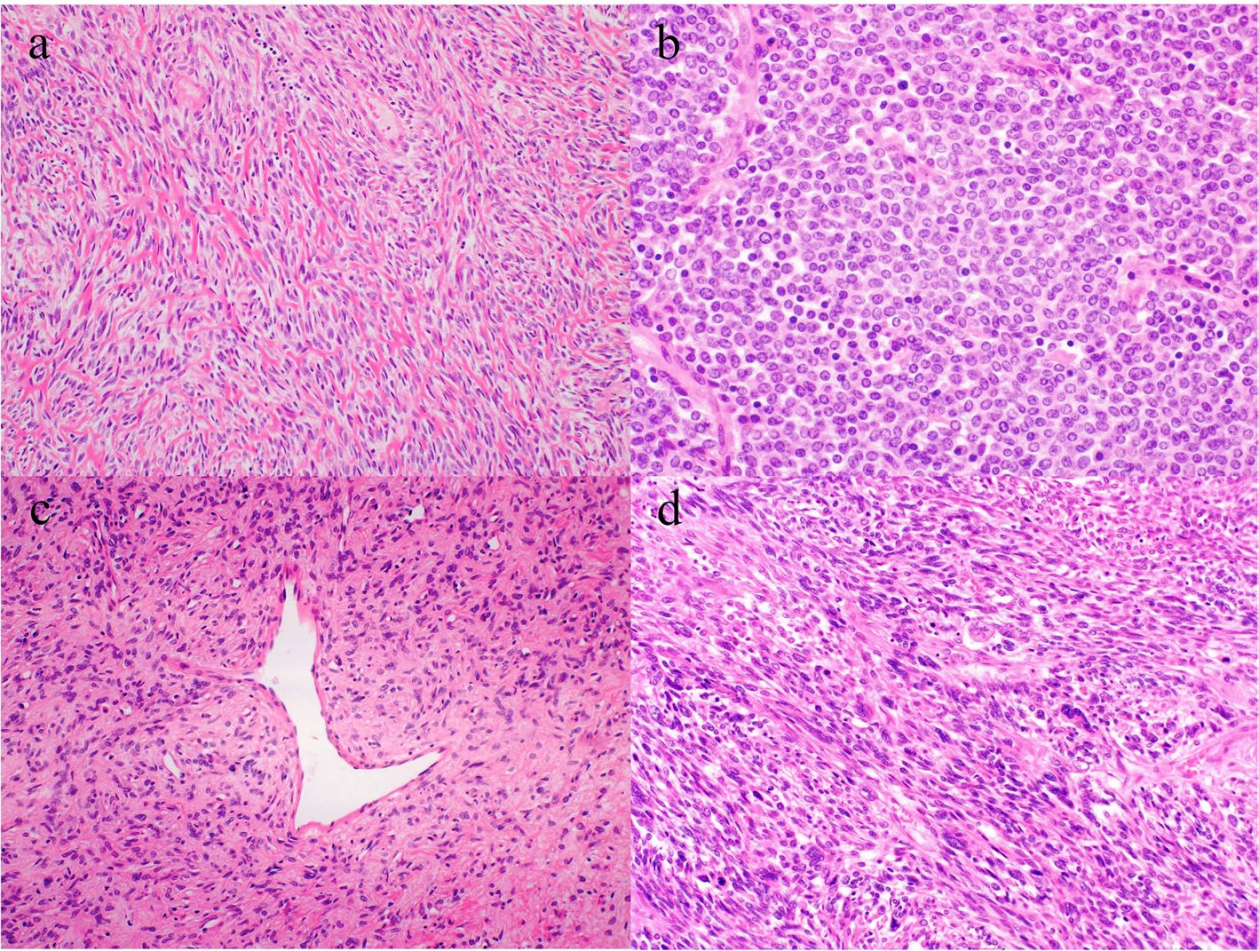
Histological features of conventional and dedifferentiated solitary fibrous tumors. **a** Solitary fibrous tumor composed of a fascicular or haphazard proliferation of spindle to oval cells with bland oval to spindle nuclei, pale eosinophilic cytoplasm. and abundant collagenous stroma. **b** A tumor composed of a solid proliferation of round to epithelioid cells with increased cellularity and less collagenous stroma. **c** So-called hemangiopericytoma-like vasculature. **d** Dedifferentiated areas showing a high-grade sarcoma component composed of anaplastic cells with severe nuclear atypia

There were 41 conventional SFTs and two dedifferentiated SFTs. There were no fat-forming or giant cell-rich variants. Histologically, the conventional SFTs consisted of a fascicular or haphazard proliferation of spindle to oval cells that contained bland oval to spindle nuclei and pale eosinophilic cytoplasm (Fig. 1a). The tumors often had abundant collagenous stroma. Some tumors consisted of a solid proliferation of round to epithelioid cells with increased cellularity and less collagenous stroma (Fig. 1b). The so-called hemangiopericytoma-like vasculature was observed and with occasional hyalinization of the blood vessel wall (Fig. 1c). However, both the dedifferentiated SFTs showed an abrupt transition between conventional and dedifferentiated areas. The dedifferentiated areas contained a high-grade sarcoma component that was composed of anaplastic cells with severe nuclear atypia (Fig. 1d). No foci of heterogeneous differentiation were found in the dedifferentiated components.

### Immunohistochemistry

**Fig. 2:**
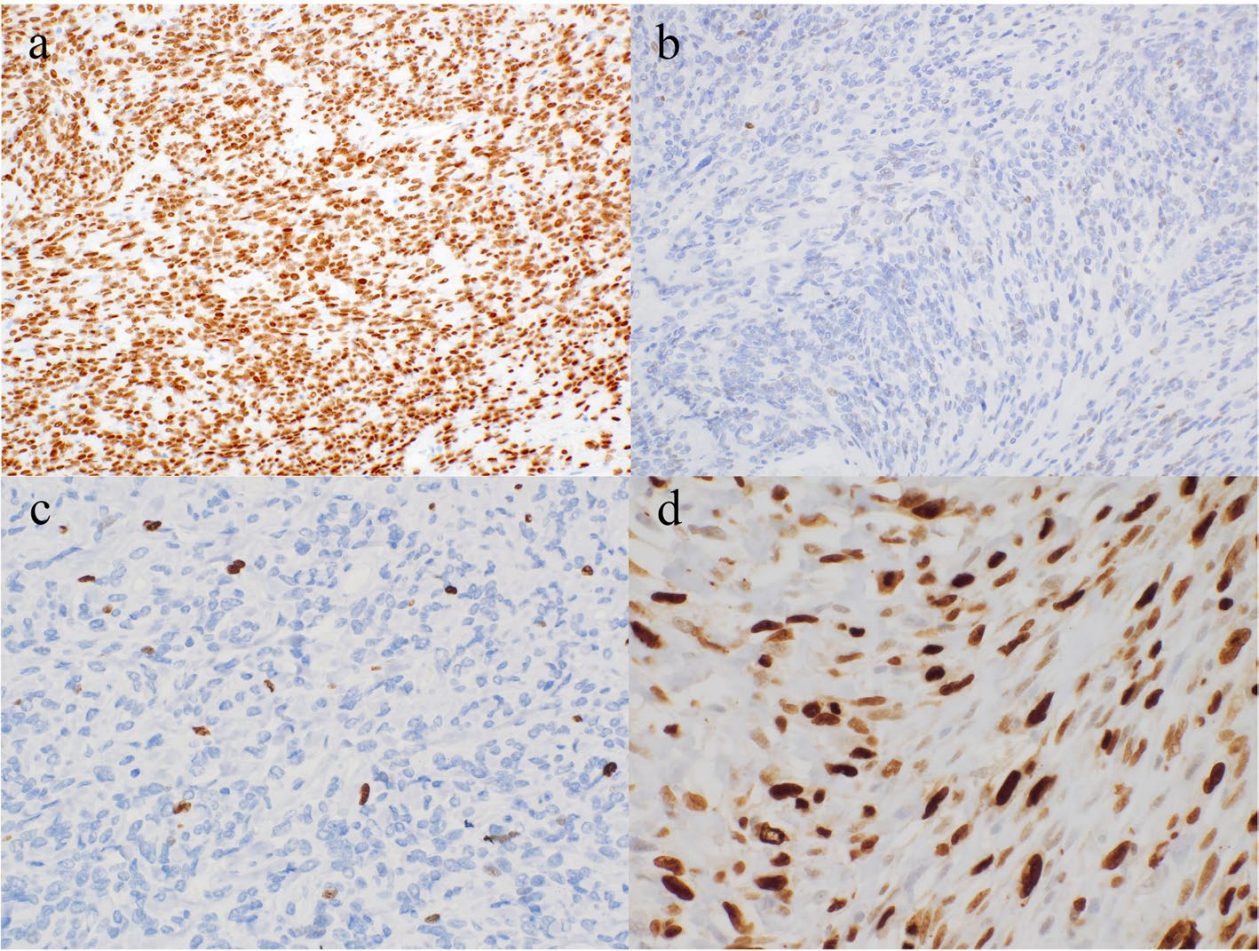
Immunohistochemical findings in conventional and dedifferentiated SFTs. **a** Tumor showing diffuse and strong nuclear expression of STAT6. **b** Tumor cells with no STAT6 expression in dedifferentiated areas. **c** Tumor showing a variable degree of Ki-67 positivity. Many areas had a low Ki-67 labeling index. **d** Tumor cells in dedifferentiated areas showing a high Ki-67 labeling index

On IHC, all SFTs showed a variable degree of STAT6 positivity in the tumor nuclei. Many exhibited diffuse and strong nuclear expression of STAT6 (Fig. 2a). The two cases of dedifferentiated SFT showed nuclear STAT6 expression in the conventional SFT area but no STAT6 expression (Fig. 2b) or CD34 expression (data not shown) in the dedifferentiated areas. All SFTs showed some degree of Ki-67 positivity (Fig. 2c). Using the MCI method and image analysis software, we found that the Ki-67 LI of the tumor cells ranged from less than 1% to as high as 72%. The two dedifferentiated SFTs showed markedly high Ki-67 LI (66% and 72%) in the dedifferentiated areas (Fig. 2d).

### Clinicopathological parameters included in the risk models for SFT

The clinicopathological parameters included in the risk models are summarized in Table 1. For age, 19 of the 43 patients had a score of 0 (< 55 years) and 24 had a score of 1 (≥ 55 years). For tumor size, 24 patients had a score of 0 (0–4.9 cm), 10 had a score of 1 (5–9.9 cm), 3 had a score of 2 (10–14.9 cm), and 6 had a score of 3 (≥ 15 cm). For mitotic frequency, 34 patients had a score of 0 (0/mm^2^), 4 had a score of 1 (0.5–1.5/mm^2^), and 5 had a score of 2 (≥ 2/mm^2^). For tumor necrosis, 40 patients had a score of 0 (< 10%) and three had a score of 1 (≥ 10%). For Ki-67 LI, 15 patients had a score of 0 (< 1%), 24 had a score of 1 (1%–10%), and 4 had a score of 3 (≥ 10%).

### Statistical analysis

**Fig. 3:**
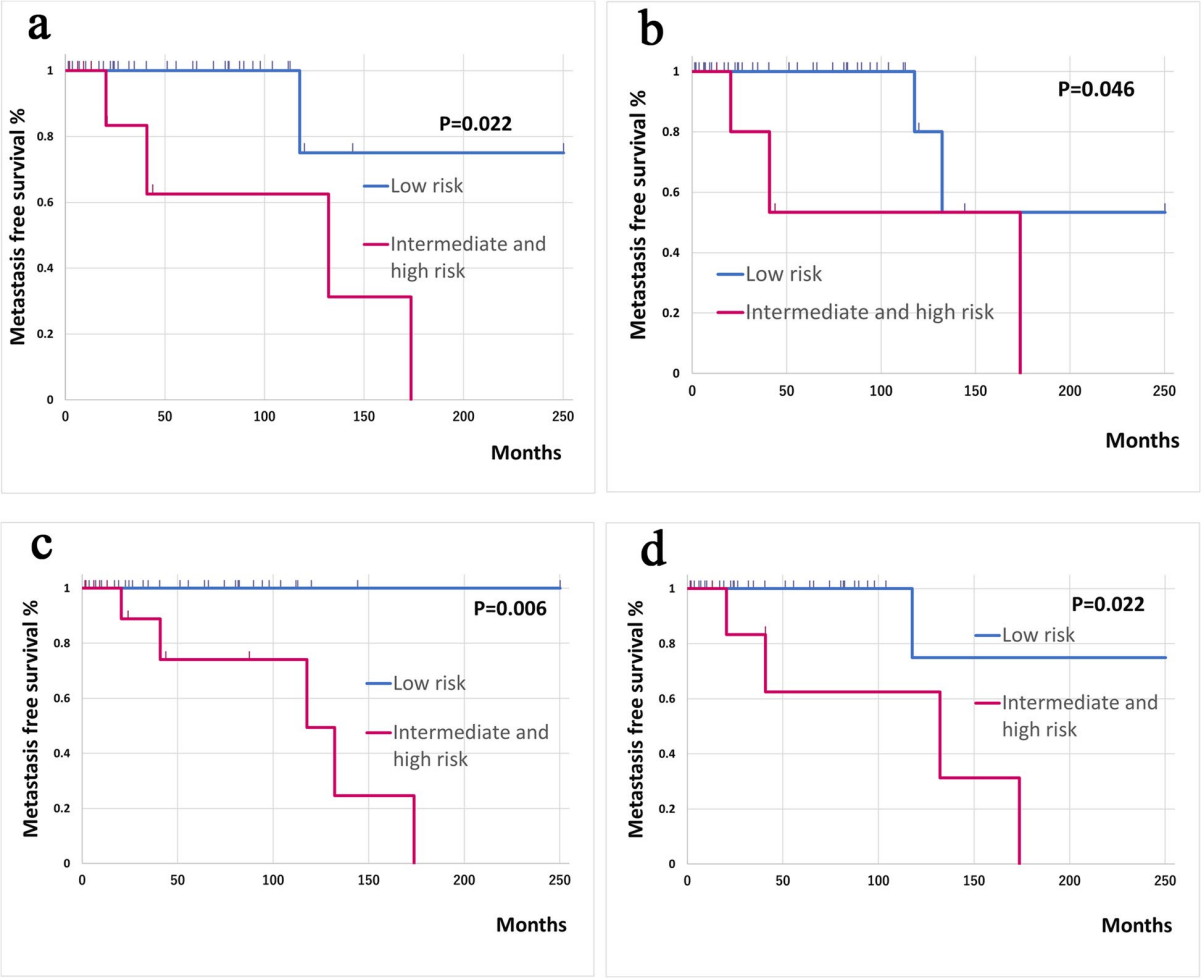
Kaplan–Meier curve analysis for MFS using the different risk models. There was a significant difference in MFS between the low-risk group and intermediate/high-risk group using the **a** three-variable Demicco risk model (*P* = 0.022) and **b** four-variable Demicco risk model (*P* = 0.046). There was also a significant difference in MFS between the low-risk group and intermediate/high-risk group using the modified **c** three-variable (*P* = 0.006) and **d** four-variable (*P* = 0.022) risk models in which mitotic count was replaced with the Ki-67 labeling index

Kaplan–Meier curve analysis was performed to compare the performance of the three-variable and four-variable risk models proposed by Demicco et al. with that of our modified risk models that replaced the parameter of mitotic count with the Ki-67 LI. There was no significant difference in metastasis-free survival (MFS) when patients were categorized into three risk groups (low, intermediate, and high). However, when patients were classified into only two risk groups (low and intermediate/high), there was a significant difference in MFS using both the three-variable (*P* = 0.022; Fig. 3a) and four-variable (*P* = 0.046; Fig. 3b) Demicco models. Furthermore, there was a significant difference in MFS between these two risk groups when our modified three-variable model (*P* = 0.006; Fig. 3c) and four-variable model (*P* = 0.022; Fig. 3d) were used.

## Discussion

Various clinicopathological factors are thought to be associated with the prognosis in patients with SFT [14, 15]. The histological criteria for malignancy in these patients have been based mainly on mitotic activity (> 4/10 high-power fields) [4]. Increased cellularity, nuclear atypia, and pleomorphism are not always associated with the outcome. Moreover, several clinical parameters, including patient age, anatomical location, tumor size, and treatment-related factors, have also been explored as potential predictors of the prognosis. Therefore, a risk classification that combines these parameters has been proposed [5, 6]. However, a recent study by Yamada et al. demonstrated that mortality was positively associated with male sex, larger tumor size, hypoglycemia, and dedifferentiation [16]. Moreover, multivariate analysis revealed that dedifferentiation was an independent predictor of overall survival. Indeed, most pathologists can easily recognize dedifferentiated SFT, which contains areas of typically diffuse hypercellularity and lacks the morphologic features of conventional SFT. As seen in our cases, dedifferentiated areas are usually demarcated from areas of conventional SFT, and high-grade areas do not have the typical staining patterns of CD34 and STAT6 associated with conventional SFT on IHC. However, it can sometimes be difficult to distinguish dedifferentiated SFT from conventional SFT with areas showing malignant features, such as epithelioid or round cell morphology, high mitotic activity, hemorrhage, and necrosis. Some cases of dedifferentiated SFT may retain STAT6 expression or stain heterogeneously for STAT6 on IHC [17]. Moreover, in our series, there were some patients with conventional SFT without dedifferentiation who developed metastasis. Therefore, we sought to identify morphological and IHC parameters other than dedifferentiation that could predict the prognosis of SFT.

First, we investigated the value of the Demicco risk models in our cohort. The cohort was too small to be able to demonstrate a significant difference in MFS between low-risk, intermediate-risk, and high-risk groups; however, there was a significant difference in MFS when the patients were divided into only two groups (low risk vs intermediate/high risk). The Demicco method may be more useful for predicting MFS in such a small cohort.

Mitotic count is included in some sarcoma grading systems, including the FNCLCC grading method, which has been widely used to grade sarcoma [7]. Mitotic count is an important morphological parameter when assessing the proliferative activity of tumor cells. However, evaluation of the mitotic count may vary from observer to observer [8]. Many factors contribute to interobserver differences in mitotic counts, including inaccurate criteria used for identification of mitotic figures, quality of tissue processing, and selection of the counting area. Therefore, we have used the Ki-67 LI instead of the mitotic count to grade sarcoma and found it to be useful for predicting the prognosis of patients with sarcoma [9–12]. Ki-67 LI can quantify the proliferative potential of tumor cells, and measurement of Ki-67 with image analysis can make the evaluation more universally applicable [13]. In this study, we substituted mitotic count for the Ki-67 LI in the Demicco risk models and found a significant difference in MFS between a low-risk group and an intermediate/high risk group. The Ki-67 LI is useful for predicting the prognosis of patients with soft tissue tumors, including SFT [8], and modified three-variable or four-variable risk models that include Ki-67 LI are considered to be more objective and nonrestrictive than the risk model based on mitotic count.

It is described that a multidisciplinary approach including surgery is important for the management of retroperitoneal sarcoma including SFT [18]. There is also a report that invasive breast cancer and malignant mediastinal SFT occurred in a patient with Li-Fraumeni syndrome, *TP53* mutation was detected in lung metastatic lesions of SFT, and the rare *TP53* variant was involved in tumor progression [19]. Thus, needless to say many factors other than Ki-67 LI can affect the prognosis of SFT.

## Conclusion

Our modified risk models using Ki-67 LI were confirmed to be effective tools for prediction of the prognosis of patients with SFT.

## Data Availability

All data were presented in this paper and there were no additional supporting files.

## Abbreviations

SFT: Solitary fibrous tumor
IHC: Immunohistochemistry
FNCLCC: Federation Nationale des Centres de Lutte le Cancer
MCI: Manually captured image
Ki-67 LI: Ki-67 labeling index
